# Effect of Ivermectin^®^ on survivorship and fertility of *Anopheles arabiensis* in Ethiopia: an in vitro study

**DOI:** 10.1186/s12936-023-04440-6

**Published:** 2023-01-09

**Authors:** Kasahun Eba, Tibebu Habtewold, Lechisa Asefa, Teshome Degefa, Delenasaw Yewhalaw, Luc Duchateau

**Affiliations:** 1 Department of Environmental Health Science and Technology, Jimma University, P.O. Box 378, Jimma, Ethiopia; 2 Department of Life Sciences, Imperial College London, London, UK; 3 Department of Environmental Health Sciences, Bule Hora University, P.O. Box 144, Bule Hora, Ethiopia; 4 School of Medical Laboratory Sciences, Jimma University, P.O. Box 378, Jimma, Ethiopia; 5 Tropical and Infectious Diseases Research Center, Jimma University, P.O. Box 378, Jimma, Ethiopia; 6 Biometrics Research Center, Faculty of Veterinary Medicine, Ghent University, Ghent, Belgium

**Keywords:** Ivermectin, Mosquito mortality, *An. arabiensis*, Wild mosquitoes, Laboratory mosquitoes, Ethiopia

## Abstract

**Background:**

Innovative vector control tools are needed to counteract insecticide resistance and residual malaria transmission. One of such innovative methods is an ivermectin (IVM) treatment to reduce vector survival. In this study, a laboratory experiment was conducted to investigate the effect of ivermectin on survivorship, fertility and egg hatchability rate of *Anopheles arabiensis* in Ethiopia.

**Methods:**

An in vitro experiment was conducted using 3–5 days old *An. arabiensis* adults from a colony maintained at insectary of Tropical and Infectious Diseases Research Center, Jimma University (laboratory population) and *Anopheles* mosquitoes reared from larvae collected from natural mosquito breeding sites (wild population). The mosquitoes were allowed to feed on cattle blood treated with different doses of ivermectin (0 ng/ml, 5 ng/ml, 10 ng/ml, 20 ng/ml, 40 ng/ml and 80 ng/ml). During each feeding experiment, the mosquitoes were held in cages and blood-fed using a Hemotek feeder. Mortality and egg production were then recorded daily for up to 9 days. Time to death was analysed by a Cox frailty model with replicate as frailty term and source of mosquito (wild versus laboratory), treatment type (ivermectin vs control) and their interaction as categorical fixed effects. Kaplan Meier curves were plotted separately for wild and laboratory populations for a visual interpretation of mosquito survival as a function of treatment.

**Results:**

Both mosquito source and treatment had a significant effect on survival (*P* < 0.001), but their interaction was not significant (*P* = 0.197). Compared to the controls, the death hazard of *An. arabiensis* that fed on ivermectin-treated blood was 2.3, 3.5, 6.5, 11.5 and 17.9 times that of the control for the 5 ng/ml, 10 ng/ml, 20 ng/ml, 40 ng/ml, and 80 ng/ml dose, respectively. With respect to the number of hatched larvae, hatched pupae and emerged adults per fed mosquitoes, a significant difference was found between the control and the 5 ng/ml dose group (*P* < 0.001). The number of hatched larvae and pupae, and emerged adults decreased further for the 10 ng/ml dose group and falls to zero for the higher doses.

**Conclusion:**

Treating cattle blood with ivermectin reduced mosquito survival, fertility, egg hatchability, larval development and adult emergence of *An. arabiensis* in all tested concentrations of ivermectin in both the wild and laboratory populations. Thus, ivermectin application in cattle could be used as a supplementary vector control method to tackle residual malaria transmission and ultimately achieve malaria elimination in Ethiopia.

## Background

Impressive progress has been made globally to control malaria over the last two decades. The malaria case incidence rate (number of cases per 1000 population at risk) has decreased from 80 in 2000 to 57 in 2019, a reduction of almost 30% [1]. The mortality incidence rate (number of deaths per 100,000 population at risk) reduced from 25 in 2000 to 10 in 2019, or a reduction of 60% [1]. Over the same period, the number of countries with fewer than 100 indigenous malaria cases increased from 6 to 27, with 21 countries reporting zero malaria cases for at least three consecutive years, and 10 of these countries were certified malaria-free by the World Health Organization (WHO) [1]. Similarly, malaria trend declined over the last 15 years in Ethiopia, mainly as a result of intensive use of control interventions, such as artemisinin-based combination therapy (ACT), use of rapid diagnostic tests (RDTs) at the remote health facilities, wide-scale distribution of long-lasting insecticidal nets (LLINs) and increased coverage of indoor residual spraying (IRS) since 2004/2005 [2, 3]. However, malaria still remains a major public health problem in the country [4].

Malaria vector control tools such as LLINs and IRS have played a significant role in reducing the burden of malaria in Africa by targeting mosquitoes that feed on human indoors (anthropophilic, endophagic), and rest inside houses (endophilic) [5, 6]. These indoor interventions reduce the feeding frequency, density, and survival of endophagic and endophilic vector species, such as *Anopheles gambiae* and *Anopheles funestus* [7]. However, their progress is limited by residual transmission, through mosquitoes that rest outside after feeding, as well as those that avoid contact with these indoor vector control strategies [8]. The impact of IRS on *An. arabiensis* has decreased, partly due to the exophilic and zoophilic nature of these mosquitoes [9]. Thus, transmission risk and burden of malaria in Africa is still high even in areas with high coverage of LLINs and IRS [10, 11], where malaria vectors exhibit zoophagic, exophagic and exophilic characteristics, such as in *An. arabiensis* [12, 13]. Moreover, the transmission risk is high in settings where insecticide resistance builds up [14, 15],

*Anopheles arabiensis* continues to play a significant role in malaria transmission in East African countries [16], where it frequently feeds on cattle and on unprotected humans outdoors to sustain residual malaria transmission [17, 18]. Studies conducted in East Africa documented that there is a shift in vector species composition from predominantly endophagic *An. gambiae *sensu stricto (*s.s*.) to predominantly exophagic *An. arabiensis* following the scale-up of ITNs [10, 19].

A study documented that malaria transmission considerably reduced in Ethiopia as a result of available malaria vector control tools [20]. However, insecticide resistance and the change in behaviour of *Anopheles* mosquitoes remain a challenge for the effectiveness of malaria control tools [21, 22]. Thus, to achieve malaria elimination, innovation of vector control tools to counteract the emergence of drug and insecticide resistance is fundamental [23]. Much of the success made in reducing the malaria burden has been due to control of mosquito vectors. In line with this, previous studies suggested additional vector control tools such as use of endectocides, zooprophylaxis, improving housing, odour-baited mosquito trapping systems and larval control measures as potential alternatives [24–26]. These alternative are important to be used in settings where there is high risk of residual malaria transmission, insecticide resistance and asymptomatic malaria to achieve and sustain zero malaria transmission [27].

Ivermectin was the world’s first endectocide, able to kill diverse endo-and ecto-parasitic nematodes and arthropods [28] and is a deep seated veterinary endectocide, first approved in 1987 to use against onchocerciasis [29]. Besides its broad anti-parasitic activity against onchocerciasis and lymphatic filariasis, ivermectin has been found to be effective in killing mosquitoes that feed on treated humans and livestock [30–32]. Ivermectin primarily targets the glutamate-gated chloride channel and it constitutes a different mode of action to insecticides currently available on the public health market [33]. In addition, ivermectin is different from LLINs and IRS in a way that it targets mosquitoes that bite and rests outdoors. [34].

Studies documented that ivermectin remains in the human blood stream following a standard oral dose and can kill blood-feeding *Anopheles* [35, 36] A relatively small concentration of ivermectin can kill the mosquito before they become infectious was documented by a previous study [37]. *Anopheles arabiensis*, is an opportunistic feeder showing flexibility in both resting and feeding habits [38]. Thus, the zoophagic behaviour of vectors may be an opportunity to use ivermectin-treated animals to kill mosquitoes.

However, there is limited information in Ethiopia on the use of ivermectin as a strategy to reduce the burden of malaria using ivermectin-treated cattle blood. Specifically, the lethal and sublethal effect of ivermectin on *An. arabiensis* fed on ivermectin-treated cattle blood is unknown. Thus, in this study a laboratory experiment was conducted to investigate the effect of ivermectin-treated cattle blood on survivorship, fertility and egg hatchability rate of *An. arabiensis*.

## Methods

### Colony of *Anopheles arabiensis*

The experiment was conducted using 3–5 days old *An. arabiensis* adults from the colony maintained at the insectary of Tropical and Infectious Diseases Research Center (TIDRC) of Jimma University. The *An. arabiensis* mosquitoes (Sekoru strain colonized from Adama, Ethiopia) were reared in the insectary by maintaining standard insectary conditions i.e., temperature of 27 ± 2 °C and relative humidity of 75 ± 10% for adults, and temperature of 31 ± 2 °C for larvae room. Rabbits were used to feed the adults in the insectary.

### Wild mosquito rearing in the laboratory

*Anopheles* mosquito larvae were collected by dipping from natural mosquito breeding sites located in the Gilgel Gibe watershed, southwest Ethiopia. During the collection, larvae were placed in plastic containers (3 L) half-filled with water from the same breeding site and transported to the Jimma University TIDRC. The larvae were provided with a diet of ground Tetramin^®^ fish food. Upon emergence, pupae were collected in cups (200 ml) and placed in a 30 cm × 30 cm × 30 cm cage for 3 days until they emerged to adults. The emerged mosquitoes were identified morphologically and only female *An. gambiae *sensu lato (*s.l*.) mosquitoes were used for the experiment after 3–5 days maturation.

### Ivermectin solution preparation

An ivermectin stock solution of 10 mg/ml, commercially available, was obtained from Jimma University Veterinary clinic. Ivermectin solution was prepared from the stock solution of (10 mg/ml) as follows:

1. Diluting the initial ivermectin stock solution 100× (10 ul of ivermectin + 990 ul of distilled water). This gives a concentration of 0.1 mg/ml or 100,000 ng/ml. This was considered as Dilution 1.
2. Then, the final concentration was prepared as follows:

**Table:** 

Volume of Ivermectin solution from Dilution 1	0	1 µl	2 µl	4 µl
Volume of cattle whole blood	5 ml	5 ml	5 ml	5 ml
Final concentration	0 ng/ml	20 ng/ml	40 ng/ml	80 ng/ml

### Mosquito blood-feeding

Cattle blood was collected daily from a local abattoir and used for all blood feeds. Prior to the experiment, 3–5 day-old female *An. gambiae s.l.* mosquitoes were starved of sugar solution for 8 h. Each cattle blood sample with a particular dose of ivermectin was presented to a batch of 50 females *An. gambiae s.l.* (corresponding to the experimental unit) in triplicate. The mosquitoes were held in 30 cm × 30 cm × 30 cm cages and offered the blood using a Hemotek feeder (Discovery Workshops, Accrington, UK) (placed on the upper surface) covered with collagen membrane and heated to 37 °C. Mosquitoes were allowed to feed for approximately 30 min.

### Mosquito mortality

After blood feeding, unfed mosquitoes were removed from the cage using a mouth aspirator and transferred to paper cups, and the total numbers of blood-fed mosquitoes were recorded. Mortality was recorded every 24 h for 9 days post blood-feeding, with dead mosquitoes removed daily. New batches of *gambiae s.l* were used for each replicate. Throughout the study, adult mosquitoes were provided with cotton moistened with 10% sugar solution. Mosquitoes were considered dead if they were lying on the bottom of the cage and unable to move. The mosquito that is unable to fly but able to stand on its leg was recorded as alive. All dead wild *An. gambiae s.l.* were preserved individually in labelled 1.5 ml Eppendorf tubes containing silica gel desiccant, and stored at − 20 °C freezer at the TIDRC laboratory for further molecular identification.

### Egg production and developmental stages

To assess the impact of the ivermectin dose on the number of eggs produced from surviving females, petri dishes lined with cotton batting, a filter paper, and moistened with deionized water were placed in each cage as an oviposition substrate [39]. The filter papers were replaced daily starting from 3 to 5 days post blood feeding [37, 40, 41]. The laid eggs were counted and recorded daily and then placed in plastic pans filled with distilled water. Room temperature was maintained at 30 °C for the hatchability test. The eggs were counted using dissecting microscope at an ocular magnification of 10x. Laid eggs were reared in separate plastic cups filled with distilled water. The number of newly emerged larvae was recorded. The hatched larvae were provided with instant dry yeast daily in larval pans and their development to pupae was observed for 7 days. The emerged pupae were collected in cups and placed in another 30 cm × 30 cm × 30 cm cage to examine the emergence to adults for 3 days [42, 43]. After the experiment, all surviving wild female mosquitoes initially introduced into the gages were preserved individually in 1.5 ml Eppendorf tubes for further molecular identification.

### Identification of vector species complexes

Sub-samples of the wild caught *An. gambiae s.l.* specimens used for the experiment were analysed by polymerase chain reaction (PCR) for identification of their sibling species, following the protocol developed by Scott et al. [44].

### Data analysis

Time to death was analysed by a Cox frailty model with replicate as frailty term and source of mosquito (wild versus laboratory), treatment and their interaction as categorical fixed effects. Kaplan Meier curves are provided separately for wild and laboratory mosquitoes for a visual interpretation of mosquito survival as a function of treatment.

The other variables, percentage of fed mosquitoes that lay eggs, number of eggs per fed mosquito, number of hatched larvae per fed mosquito, number of hatched pupae per fed mosquito and number of emerged adults per fed mosquito were analysed by a mixed model with replication as random effect and source of mosquito (wild versus laboratory), treatment and their interaction as categorical fixed effects. For this analysis, only control and the lower doses were considered, as these reproduction parameters were zero for the three highest doses and for most response variables for the second lowest dose.

## Results

### Mosquito species composition

A total of 350 wild *An. gambiae s.l.* specimens used for the experiment were analysed for sibling species identification using PCR. The PCR results revealed that all *An. gambiae s.l.* specimens were found to be *An. arabiensis*.

### Feeding rate

The number of *An. arabiensis* exposed to feed on cattle blood using membrane feeder were 3000 and out of this 2007 mosquitoes became fully engorged. The feeding rate of mosquitoes was 66.9%. There were no significant differences in mosquito feeding rates between treatment and control groups (65.72% and 68.2% average feeding rates, respectively, *P* = 0.178).

### Mosquito mortality

**Fig. 1:**
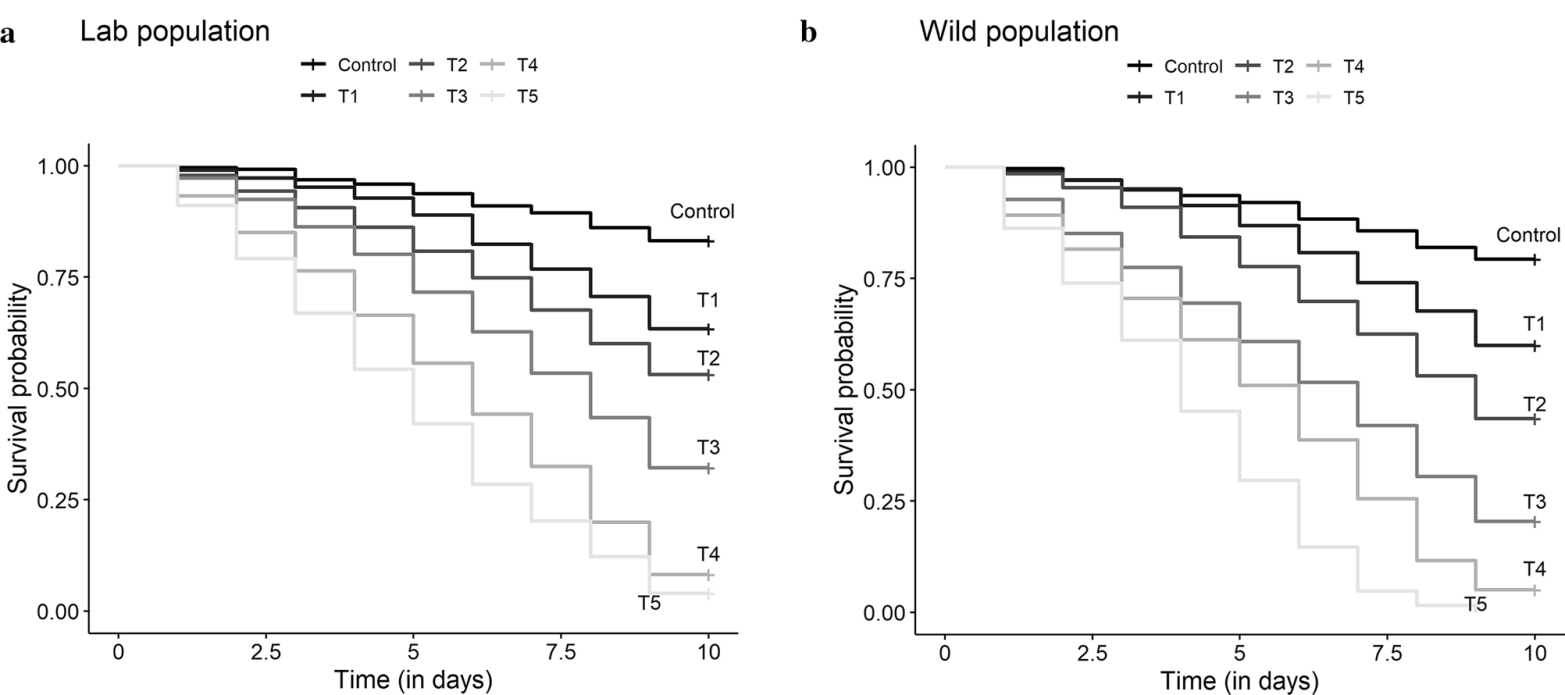
**a** Survival curves of the laboratory mosquitoes as a function of the Ivermectin dose. (Control), 5 (T1), 10 (T2), 20 (T3), 40 (T4) and 80 (T5) ng/ml ivermectin. **b** Survival curves of the wild mosquitoes as a function of the Ivermectin dose. 0 (Control), 5 (T1), 10 (T2), 20 (T3), 40 (T4) and 80 (T5) ng/ml Ivermectin

With respect to mosquito mortality, there was a significant effect of both source (laboratory and wild mosquitoes) (P < 0.001) and treatment (P < 0.001), but the interaction was not significant (P = 0.197). The hazard ratios of mosquito mortality in comparison with control were 2.30 (95% CI [1.90;2.77]) for the 5 ng/ml dose, 3.52 (95% CI [2.96;4.19]) for the 10 ng/ml dose, 6.51 (95% CI [5.50;7.70]) for the 20 ng/ml dose, 11.47 (95% CI [9.76;13.48]) for the 40 ng/ml dose and 17.89 (95% CI [15.19;21.06]) for the 80 ng/ml dose (Fig. 1a and b). A monotone increase in the hazard ratio as a function of the ivermectin concentration was observed. The hazard ratio for wild mosquitoes was 1.33 (95% CI [1.23; 1.44]) compared to the laboratory mosquitoes. Survival was high in the control throughout, while the survival of *An. arabiensis* mosquitoes reduced with an increasing concentration of ivermectin ingestion for the treatment groups. The death hazard of *An. arabiensis* for the 80 ng/ml dose was 18 times that of the control groups, while the death hazard for the lowest concentration of the ivermectin (5 ng/ml) was twice that of the control group.

At day 9 post-feeding, the mortality rates of *An. arabiensis* that fed on ivermectin-treated cattle blood were 69.70%, 77.88%, 86.68%, 91.98% and 94.71% at concentrations of 5 ng/ml, 10 ng/ml, 20 ng/ml, 40 ng/ml and 80 ng/ml, respectively, while the mortality rate was 12% in the control group (Fig. 1a and b).

### Effect of ivermectin on egg production, hatchability, pupae and adult emergence

With respect to the percentage of fed mosquitos that laid eggs, a significant difference was found between the control and the 5 ng/ml dose group (*P* < 0.001), with lower percentages for the last group. The percentage decreases further for the 10 ng/ml dose group and falls to zero for the higher doses (Table 1).

**Table 1:** Effect of ivermectin treated cattle blood on egg production, hatchability, pupa development and adult emergence (n = 512 for laboratory and 537 for wild populations)

Response	Source	Control	5 ng/ml	10 ng/ml	20 ng/ml	40 ng/ml	80 ng/ml
Percentage egg laying	L	94.1 (1.2)	88.9 (1.4)	80.7 (1.4)	0	0	0
	W	92.8 (1.2)	86.4 (1.4)	77.7 (1.4)	0	0	0
Av. No. eggs	L	46.9 (1.2)	18.7 (1.5)	4.9 (1.4)	0	0	0
	W	45.0 (1.2)	14.5 (1.5)	0 (0)	0	0	0
Av. No. hatched	L	30.5 (1.0)	9.9 (1.3)	0.8 (1.3)	0	0	0
	W	27.2 (1.0)	5.3 (1.3)	0 (0)	0	0	0
Av. No. pupae	L	15.5 (0.5)	3.7 (0.7)	0.3 (0.7)	0	0	0
	W	11.4 (0.5)	1.9 (0.7)	0 (0)	0	0	0
Av. No. adults	L	7.0 (0.2)	1.5 (0.3)	0.1 (0.3)	0	0	0
	W	4.7 (0.2)	0.6 (0.3)	0 (0)	0	0	0

*L* laboratory population,* W*  wild population,* Av.* average

The percentage of mosquitoes laying eggs after feeding on treated cattle blood was reduced to 77.7% for the 10 ng/ml dose compared to the control group for the wild group, while it was reduced to 80.7% for the laboratory group. Moreover, for the 10 ng/ml dose, the average number of eggs laid for the laboratory group was reduced to 4.9 compared to the average number of eggs laid by the control group, which was 46.9. Similarly, the average number of eggs laid by the wild group reduced to 0 for the same concentration (Table 1).

The number of eggs per fed mosquito was significantly lower in the 5 ng/ml dose group compared to the control group (*P* < 0.001), and also significantly lower in the wild group compared to the laboratory group (*P* = 0.029), but the two factors did not interact (Table 1).

With respect to number of hatched larvae, hatched pupae and emerged adults per fed mosquito, a significant difference was found between the control and the 5 ng/ml dose group (P < 0.001), with lower percentages for the last group. The number decreased further for the 10 ng/ml dose group and fell to zero for the higher doses. A significantly lower number was also found in the wild group compared to the laboratory group (*P* < 0.001), but the two factors did not interact (Table 1).

Larvae, pupae and adults only emerged from the lowest dose (5 ng/ml) for the wild and laboratory groups and for the 10 ng/ml dose only from the laboratory group.

## Discussion

This study demonstrated that ivermectin-treated cattle blood is able to reduce the survival, fertility, egg hatchability, larval development and adult emergence of wild and laboratory-reared *An. arabiensis* mosquitoes, at various concentrations of the drug. Survival of *An. arabiensis*, the most influential variable for vectorial capacity, was significantly reduced after the laboratory and wild mosquitoes were allowed to feed on ivermectin treated cattle blood at all concentrations. Similarly, previous studies documented that ivermectin significantly reduced the survivorship of *Anopheles* mosquitoes under laboratory conditions [33, 45–47]. Field studies showed that ivermectin was lethal to *An. arabiensis* at low concentrations [47–49]. Moreover, previous studies showed that a long-lasting formulation of ivermectin administered to calves decreased field malaria vector populations [50]. The result of this study showed that the wild mosquitoes used in this study were more susceptible to ivermectin than laboratory mosquitoes. The variation in susceptibility of wild and laboratory mosquitoes to ivermectin is not known as there might be difference in exposure to insecticides and genetic diversity. Moreover, the laboratory mosquitoes are intercrossed and do not represent the mosquitoes in the field. Similarly, a previous study reported that in a laboratory setting, it took several days for laboratory-reared *An. gambiae* to respond to a lethal blood meal. [51]. Moreover, a previous study showed the field population of *An. arabiensis* was more susceptible to clothianidin compared to the laboratory strain [52].

In the present study, the concentration of ivermectin in cattle blood at 80 ng/ml increased the hazard of death of *An. arabiensis* by 18-fold relative to control. In addition, the current study revealed a monotone increase in the hazard ratio as a function of the ivermectin concentration for both laboratory and wild groups. Similarly, previous studies revealed that increasing the concentration of ivermectin reduced survival of *An. arabiensis* [49] and increased the risk of death of mosquitoes five-fold relative to control [32]. Moreover, the result of this study supports earlier observations [53, 54]. Thus, this confirms that ivermectin can alter the most influential variable for vectorial capacity, the daily probability of adult survivorship [55–58].

The current study revealed that no eggs were laid by the mosquitoes that fed on ivermectin treated cattle blood at concentrations larger than 10 ng/ml. Moreover, the number of eggs laid by *An. arabiensis* mosquitoes was significantly reduced after feeding on ivermectin-treated cattle blood at any dose. A previous study also documented that the probability that *An. arabiensis* lay eggs after blood feeding was significantly affected by ivermectin-treated cattle [32]. Similarly, previous studies conducted on different principal malaria vectors documented a similar effect of ivermectin on mosquito fecundity [39, 43, 53, 59, 60]. Moreover, a study has shown that blood meal digestion in the stomach of *An. arabiensis* is reduced when fed on ivermectin-treated cattle and hence egg production [32]. On the other hand, the number of eggs laid by *Anopheles stephensi* after being fed on ivermectin-treated blood with similar concentrations as in the present study increased compared to control [41]. This might be due to difference in species with varying levels of susceptibility to ivermectin.

In the present study, the egg hatchability rate of mosquitoes fed on ivermectin-treated cattle blood was significantly reduced compared to the control group, consistent with previous studies [41, 54, 61]. This finding supports the strategy to use ivermectin in cattle as a supplementary approach to prevent malaria transmission by reducing the population of *Anopheles* mosquitoes [62, 63]. Moreover, the findings reinforce the hypothesis that even sub-lethal doses of ivermectin could play an important role in reducing the vectorial capacity of mosquitoes [63].

The present study also revealed that the median survival time of *An. arabiensis* was significantly shorter in treatment groups compared with control. The finding of this study is in line with previous studies [32, 37, 47, 64], and among treatment groups, the survival time was further reduced with increasing concentration. A previous study also revealed that increasing the ivermectin concentration reduces the mosquito median survival time [65]. Ivermectin has an impact of decreasing survival of malaria vectors which supports killing of mosquitoes before their next blood meal and becoming infectious [37].

## Conclusion

The present study demonstrated that ivermectin has the potential to reduce mosquito survival, fertility, egg hatchability, larval development and adult emergence of *An. arabiensis*. Ivermectin could thus be used as a supplementary malaria control strategy to reduce malaria transmission through targeting. Moreover, ivermectin could be useful to target outdoor feeding and resting vectors which the current available control tools fail to control. Thus, ivermectin could be used as a supplementary vector control to tackle residual malaria transmission, which is one of the current challenges in the malaria elimination phase in Ethiopia. Further field trials on the effectiveness of ivermectin in reducing mosquito vector density of *An. arabiensis*, the principal malaria vector in Ethiopia and malaria transmission intensity is recommended.

## Data Availability

Data are available from the corresponding author upon request.
